# Successful Application of Transnasal Humidified Rapid-Insufflation Ventilatory Exchange in a Case of Subglottic Stenosis

**DOI:** 10.7759/cureus.58050

**Published:** 2024-04-11

**Authors:** Cristiana Roma, Andreia Sá, Leonor Lemos, Rita Frada, Carlos Mexedo

**Affiliations:** 1 Anesthesiology and Critical Care, Unidade Local de Saúde de Santo António, Porto, PRT

**Keywords:** transnasal humidified rapid-insufflation ventilatory exchange, subglottic stenosis, microlaryngeal surgery, ent surgery, airway management

## Abstract

We widely employ microlaryngeal surgery to treat diverse oropharyngeal and laryngeal conditions, but it presents challenges to shared airway management. This case report addresses the limitations of conventional techniques, such as tracheal intubation and jet ventilation, and explores the emerging interest in transnasal humidified rapid insufflation ventilatory exchange (THRIVE). While THRIVE offers advantages such as enhanced visualization and reduced airway trauma, its application is limited by the duration of apnea, with the literature referring to a maximum of 30 minutes of apnea. We present the successful application of THRIVE as the primary airway management technique in a patient undergoing a 55-minute dilation procedure for subglottic stenosis. Successful oxygenation was achieved, creating a tubeless field and improving visibility. The patient maintained oxygen saturation above 98%, demonstrating the effectiveness of THRIVE in managing prolonged apnea. Remarkably, intentional ventilation via a face mask at specific moments allowed extended apneic oxygenation without harmful carbon dioxide levels. This report complies with the growing evidence supporting the efficacy of THRIVE in providing extended apnea for tubeless surgery. The success demonstrated in our case highlights the feasibility and effectiveness of THRIVE in situations demanding prolonged apnea and complex airway management.

## Introduction

Subglottic stenosis is a condition that can affect individuals of all age groups, presenting with a spectrum of symptoms that can vary from mild discomfort to potentially life-threatening airway obstruction. While congenital and acquired etiologies contribute to its development, acquired subglottic stenosis often arises from factors such as prior intubation, tracheostomy, radiation, autoimmune or inflammatory diseases, and trauma. The most classic examination finding is stridor, which may occur during inspiration or expiration, irrespective of the underlying cause [1].

Management strategies range from observation for mild cases to more interventional approaches for severe cases. These interventions include surgical procedures such as dilation, stent placement, laryngotracheal reconstruction, or tracheostomy [1]. Given the potential for progressive airway obstruction, timely intervention is crucial in mitigating the risks associated with this potentially life-threatening condition.

Microlaryngeal surgery is widely employed for the treatment of subglottic stenosis. While ensuring optimal access and visualization is essential in these procedures, shared airway management poses challenges for both surgeons and anesthesiologists. Despite the availability of various airway management techniques, a universally accepted gold standard has yet to be established [2].

Traditionally, tracheal intubation has been a usual practice. However, repeated extubation and reintubation to achieve a tubeless field increase the risk of barotrauma, desaturation, and potential airway injury and extend the overall surgical duration [3]. The use of jet ventilation has also been associated with certain complications, including hypoxia, hypercapnia, the necessity for rescue intubation, and barotrauma [4].

In recent years, there has been growing interest in the use of transnasal humidified rapid insufflation ventilatory exchange (THRIVE) during apnea for short laryngeal procedures. This technique employs a device capable of delivering continuous, warmed, and humidified oxygen with varying FiO_2_ levels (up to 100%) at a high flow rate (up to 70 L/min) through a high-flow nasal cannula, facilitating prolonged apneic oxygenation [5]. THRIVE presents the advantages of providing a tubeless field, improving visibility, reducing the procedure time, and mitigating endotracheal injury associated with tracheal intubation, as well as air pressure injury caused by jet ventilation [5]. Despite these advantages, its use is limited by the length of the apnea, as carbon dioxide (CO_2_) accumulation and progressive respiratory acidosis may occur during apneic periods [3,6].

In this case, we report our technique for achieving successful apneic oxygenation during a 55-minute dilation surgery to treat subglottic stenosis.

This manuscript has been previously presented in part as a poster at EUROANESTHESIA 2023 on June 4, 2023.

## Case presentation

**Figure 1:**
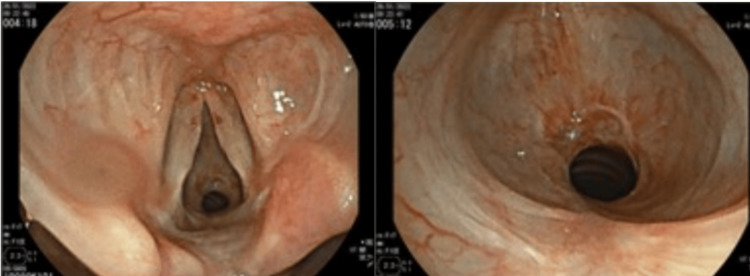
Images of our patient's subglottic stenosis.

A 60-year-old female patient with an American Society of Anesthesiology Physical Status (ASA PS) II classification due to a history of controlled hypothyroidism was scheduled for laryngotracheoscopy followed by balloon dilation of a fixed tracheal stenosis located in the subglottic region, leading to an approximately 70% reduction in tracheal diameter (Figure 1).

The patient presented with expiratory wheezing and dyspnea at rest, which had progressively worsened over the preceding month, causing difficulty in completing sentences. The onset of dyspnea occurred 30 years ago after a cesarean section that required endotracheal intubation. The condition was misdiagnosed as asthma despite normal pulmonary function tests, normal IgE levels, and unresponsiveness to asthma treatment. A definitive diagnosis of tracheal stenosis was made through bronchofibroscopy once dyspnea became evident at rest.

Anesthesia care began with pre-oxygenation using THRIVE via the Optiflow® MR810 device. The patient was instructed to spontaneously breathe with 100% oxygen delivered through a high-flow nasal cannula at a flow rate of 20 L/min for three minutes while positioned with a head elevation of 40-45°. Subsequently, the oxygen flow rate was increased to 40 L/min for an additional three minutes.

Anesthesia induction was conducted through the intravenous administration of propofol and remifentanil, according to a target-controlled infusion (Schnider and Minto models, respectively). Following neuromuscular monitor calibration, neuromuscular blockade was achieved with intravenous rocuronium. Maintenance of anesthesia involved a combination of propofol and remifentanil, with continuous monitoring of the processed electroencephalogram and maintenance of the bispectral index value within the range of 40 to 60. Standard American Society of Anesthesiologists (ASA) monitoring was supplemented with INVOS™ monitoring and intermittent arterial blood gas analysis (ABG) to assess the patient's carbon dioxide levels.

Upon the loss of consciousness, a jaw thrust maneuver with minimal mouth opening was executed to ensure an unobstructed airway and facilitate passive air egress. Simultaneously, the flow rate of high-flow nasal oxygen (HFNO) was increased to 60 L/min. Following this, the degree of head and trunk inclination was decreased to 20°, and a single ventilation via a face mask was conducted to confirm the ease of ventilation.

HFNO was consistently supplied throughout the apneic period. The anesthesiologist and surgeon developed patient-specific rescue plans if oxygenation using THRIVE proved ineffective.

The surgeon faced challenges during suspension laryngoscopy, encountering difficulties in visualizing the glottis due to the anterior positioning of the larynx. Subsequently, the surgical laryngoscope was replaced with the C-MAC® video laryngoscope to optimize the surgical view. This led to a significant extension of the initially anticipated procedure duration. Intermittent ventilation via a face mask was administered to optimize conditions and minimize the risk of severe respiratory acidosis between successive attempts at laryngoscopy. This approach aims to eliminate CO_2_ and provide additional time for surgical intervention. At this point, the arterial partial pressure of carbon dioxide (PaCO_2_) reached 76.8 mmHg, with a lactate level of 0.6 mmol/L and a pH of 7.17.

When optimal surgical visualization was achieved, the surgeon inserted a 12 mm balloon device into the airway and gently inflated it to 7-8 atm, three times for one minute each, without any desaturation occurring. After the procedure, the passage of a size 6.5 endotracheal tube was achieved without resistance, confirming the success of the procedure.

Throughout the 55-minute surgical procedure, the patient consistently maintained an oxygen saturation level above 98%. There was no hypotension associated with the elevation of PaCO_2_, and no vasopressors were required.

After the conclusion of the surgery, the face mask was used again to aid ventilation and assist with carbon dioxide elimination. Following this, the neuromuscular blockade was reversed with sugammadex, and the administration of anesthetic agents was discontinued. The patient regained consciousness and was transferred to the post-anesthesia care unit for additional monitoring and care, following our institutional protocols.

Before transferring to the ward, ABG analysis revealed a PaCO_2_ level of 53.8 mmHg, a lactate level of 0.6 mmol/L, and a pH of 7.29. The patient underwent an uneventful hospital stay and was discharged two days after the procedure.

## Discussion

Subglottic stenosis represents a complex and potentially life-threatening condition with diverse etiologies and variable clinical presentations. Stridor is a hallmark manifestation that can occur during inspiration or expiration, regardless of the underlying cause [1]. Notably, it can be misdiagnosed as asthma due to its presentation resembling expiratory wheezing, as observed in our case. Therefore, the diagnosis of subglottic stenosis should be considered in cases refractory to conventional asthma treatment, especially in patients with a history of airway instrumentation. Our case highlights the challenges associated with its management, particularly during microlaryngeal surgery, where optimal access and visualization are crucial.

THRIVE is increasingly being adopted for short laryngeal procedures as it overcomes the limitations of access and visualization of the narrowed airway and avoids the potential risks of mechanical and jet ventilation. It relies on unobstructed nasal passages to facilitate high flows; therefore, it should not be utilized in patients with severe nasal obstruction. Furthermore, it should be avoided in patients with suspected or confirmed pneumothorax, as well as in those with bullous lung disease. Additionally, THRIVE is contraindicated in patients with base of skull fractures or mid-maxillary facial trauma due to the risk of pneumocephalus and subcutaneous emphysema, respectively [7].

Beyond oxygenation, THRIVE washes out CO_2_ from anatomical dead spaces, provides unmeasured positive airway pressure, and maintains a relatively constant FiO_2_ [3]. Its efficacy relies on the patient maintaining a patent upper airway, a condition ensured by suspension laryngoscopy and neuromuscular blockade during laryngopharyngeal surgery. However, this technique is associated with a higher incidence of desaturation, hypercapnia, and the requirement for rescue intervention compared to intermittent tracheal intubation [3]. Its application is limited by the duration of apnea, as CO_2_ accumulation and progressive respiratory acidosis may occur during apneic periods, with published data indicating safety up to approximately 30 minutes [3,5]. Anesthetized individuals typically experience a decrease in systemic vascular resistance as the primary cardiovascular effect of acidosis resulting from hypercapnia [8]. Nonetheless, some studies have shown that patients can tolerate significant levels of acidosis, with some demonstrating tolerance to pH levels as low as 6.8 without exhibiting signs of physiological distress or hemodynamic compromise [9].

In our case, THRIVE was effective in providing prolonged apneic oxygenation and tubeless anesthesia during a 55-minute balloon dilation surgery under general anesthesia and neuromuscular blockade. Different from other cases reported to date [5,10], our patient was intentionally ventilated via face mask at three different moments: after induction of anesthesia to confirm ease of ventilation, when the surgeon needed to replace the laryngoscope with the C-MAC® video laryngoscope to optimize the surgical view, and after the conclusion of the surgery to facilitate ventilation and promote CO_2_ elimination. This allowed for the extension of apneic oxygenation throughout the 55-minute procedure without significant CO_2_ accumulation or hemodynamic instability. Also, our ENT surgeon was satisfied with the technique as it allowed better visualization and access and reduced airway trauma when compared with alternative ventilatory techniques.

THRIVE may still be a viable option for longer or more complex surgeries if brief ventilation via a face mask is feasible within the procedural context. Effective communication between anesthesiologists and surgeons, the implementation of individualized rescue plans, and the prompt availability of appropriate equipment are essential to addressing inadequate oxygenation and ventilation.

## Conclusions

The success demonstrated in our case highlights the feasibility and effectiveness of THRIVE in situations requiring prolonged apnea and intricate airway management. In situations involving prolonged or complex surgical procedures, THRIVE may remain a viable alternative when short-term ventilation via a face mask is feasible. However, it is crucial to establish customized rescue strategies and ensure the availability of readily accessible equipment to effectively manage instances of inadequate oxygenation and ventilation.
